# Supplementary material for: Morphological and Molecular Analysis of Australian Earwigs (Dermaptera) Points to Unique Species and Regional Endemism in the Anisolabididae Family
Source: Insects. 2019 Mar 14;10(3):72. doi: 10.3390/insects10030072 (PMC6468374; doi:10.3390/insects10030072)
Supplement: Supplementary file 1 [file insects-10-00072-s001.pdf]

insects

MDPI

Article

Morphological and Molecular Analysis of Australian Earwigs (Dermaptera) Points to Unique Species and Regional Endemism in the Anisolabididae Family

Oliver P. Stuart [1], Matthew Binns [1,2], Paul A. Umina [1,3], Joanne Holloway [4], Dustin Severtson [5], Michael Nash [6,7], Thomas Heddle [8], Maarten van Helden [6,8] and Ary A. Hoffmann [1,*]

1 School of BioSciences, Bio21 Molecular Science and Biotechnology Institute, The University of Melbourne, Parkville, Victoria 3052, Australia; oliver.stuart93@gmail.com (O.P.S.); matthew.binns@csiro.au (M.B.); pumina@unimelb.edu.au (P.A.U.)
2 Agriculture and Food Business Unit, Commonwealth Scientific and Industrial Research Organisation, Black Mountain, Australian Capital Territory 2601, Australia
3 cesar, 293 Royal Parade, Parkville, Victoria 3052, Australia
4 New South Wales Department of Primary Industries, Wagga Wagga Agricultural Institute, Pine Gully Road, Charles Sturt University, New South Wales 2795, Australia; joanne.holloway@dpi.nsw.gov.au
5 Department of Primary Industries and Regional Development, South Perth, Western Australia 6151, Australia; dustin.severtson@dpird.wa.gov.au
6 School of Agriculture, Food and Wine, the University of Adelaide, Urrbrae, South Australia 5064, Australia; Maarten.VanHelden@sa.gov.au
7 School of Life Science, College of Science, Health and Engineering, La Trobe University, Bundoora, Victoria 3086, Australia; whatbugsyou@gmail.com
8 South Australian Research and Development Institute, Entomology, Waite Road, Waite, Urrbrae, South Australia 5064, Australia; thomas.heddle@sa.gov.au
* Correspondence: ary@unimelb.edu.au

Received: 1 February 2019; Accepted: 7 March 2019; Published: date

Supplementary Materials

Table S1. Location of field sites from which adult Dermaptera were collected.

#	Site ID	State	Latitude	Longitude
1	Muradup	WA	-33.8380	116.9522
2	Frankland River	WA	-34.4123	117.0167
3	Kojonup	WA	-33.8426	117.2405
4	Mt. Barker	WA	-34.6348	117.5299
5	Tenterden	WA	-34.3549	117.6573
6	Amelup	WA	-34.3049	118.1039
7	South Stirling	WA	-34.6245	118.2758
8	Needilup	WA	-33.9553	118.7607
9	Boxwood Hill	WA	-34.4059	118.8616
10	Jacup	WA	-33.7822	119.1235
11	Fitzgerald	WA	-33.6170	119.5841
12	Wittenoom Hills	WA	-33.5208	122.2667
13	Kapinnie	SA	-34.1155	135.5122
14	Oleo Rd	SA	-34.3813	135.8071
15	North Shield	SA	-34.6090	135.8298
16	Yallunda Flat	SA	-34.3221	135.8397
17	Warooka	SA	-34.9862	137.3193
18	Petersville	SA	-34.3465	137.7843
19	Manoora	SA	-33.9520	138.8513

20	Milang	SA	-35.3834	138.9341
21	Frayville	SA	-34.8256	139.2424
22	Lake Hawdon South	SA	-37.2316	140.0259
23	Bool Lagoon	SA	-37.2049	140.7111
24	Inverleigh	VIC	-38.0862	143.9344
25	Elmore	VIC	-36.4863	144.5513
26	Finley	NSW	-35.6258	145.6040
27	Devenish	VIC	-36.3327	145.8903
28	Thoona	VIC	-36.3031	146.0989
29	Traralgon	VIC	-38.2013	146.6533
30	Grong Grong	NSW	-34.7250	146.7452
31	Downside 1	NSW	-34.9553	147.3101
32	Downside 2	NSW	-34.9574	147.3113
33	Peak Hill	NSW	-32.6696	148.0390
34	Wallendbeen	NSW	-34.5450	148.1110
35	Canowindra	NSW	-33.4256	148.6678
36	Tamworth	NSW	-31.1457	150.9742

Numbers refer to labels on Figure S1.

Table S2. Summary statistics for morphometric variables for the Anisolabididae morphospecies.

Morphospecies	N	Paramere length	Paramere width	Maximum forceps length	Maximum forceps width	Forceps asymmetry index	Paramere length-width ratio	Basal width of forceps	Forceps length-width ratio
Anisolabis sp. 1	12	1.189 (0.092)	0.396 (0.044)	3.182 (0.501)	2.476 (0.597)	0.483 (0.196)	3.027 (0.284)	3.490 (0.627)	1.320 (0.197)
		1.057 - 1.393	0.302 - 0.459	2.517 - 3.885	1.689 - 3.280	0.204 - 0.909	2.678 - 3.785	2.765 - 4.419	1.058 - 1.693
Anisolabis sp. 2	18	1.087 (0.058)	0.283 (0.027)	3.073 (0.302)	1.499 (0.150)	0.148 (0.045)	3.862 (0.354)	2.392 (0.177)	2.064 (0.241)
		0.974 - 1.198	0.229 - 0.325	2.468 - 3.633	1.190 - 1.754	0.067 - 0.219	3.361 - 4.633	1.945 - 2.624	1.750 - 2.744
Gonolabis forcipata	23	0.644 (0.034)	0.415 (0.024)	2.204 (0.260)	1.471 (0.217)	0.098 (0.040)	1.558 (0.100)	2.551 (0.280)	1.513 (0.172)
		0.583 - 0.715	0.370 - 0.466	1.717 - 2.849	1.122 - 2.049	0.047 - 0.209	1.421 - 1.806	1.987 - 3.368	1.249 - 2.041
Gonolabis nr. *gilesi*	14	0.613 (0.043)	0.353 (0.024)	1.810 (0.318)	1.402 (0.175)	0.176 (0.147)	1.744 (0.149)	2.344 (0.237)	1.302 (0.242)
		0.533 - 0.709	0.301 - 0.398	1.506 - 2.733	1.240 - 1.914	0.014 - 0.609	1.481 - 1.937	2.012 - 2.760	1.000 - 1.995
Gonolabis sp. 1	32	0.529 (0.032)	0.422 (0.026)	3.434 (0.315)	1.730 (0.272)	0.051 (0.043)	1.255 (0.074)	2.927 (0.201)	2.036 (0.394)
		0.472 - 0.590	0.369 - 0.484	2.859 - 4.116	1.276 - 2.257	0.001 - 0.174	1.129 - 1.463	2.579 - 3.320	1.498 - 2.868
Gonolabis sp. 2	22	0.658 (0.039)	0.452 (0.026)	2.469 (0.206)	1.489 (0.162)	0.078 (0.036)	1.459 (0.086)	2.827 (0.230)	1.674 (0.190)
		0.549 - 0.721	0.419 - 0.521	2.121 - 2.968	1.138 - 1.739	0.020 - 0.148	1.286 - 1.675	2.403 - 3.378	1.343 - 2.104

Gonolabis sp. 3	3	0.616 (0.084)<br>0.520 - 0.677	0.410 (0.031)<br>0.381 - 0.442	2.390 (0.431)<br>1.893 - 2.655	1.332 (0.341)<br>0.938 - 1.531	0.072 (0.011)<br>0.059 - 0.079	1.499 (0.121)<br>1.365 - 1.601	2.450 (0.468)<br>1.910 - 2.724	1.824 (0.169)<br>1.719 - 2.018

"Forceps asymmetry" is an index calculated as the sum of the absolute differences between the heights and widths of a specimen's two forceps, "Paramere ratio" is the ratio of a specimen's right paramere's length to its width, and "Forceps ratio" is the ratio of the maximum forceps length to the maximum forceps width. These three measurements were dimensionless; all others were in mm. Individual cells show the mean, the standard deviation in brackets, and the minimum and maximum values below them. N refers to the number of individuals of each morphospecies measured. Note that only the three dimensionless variables plus the basal width of the forceps were used for the morphometric and classification analysis.

Table S3. Details of linear discriminant function analysis.

Classification	Function	Variable			
		**Basal width of forceps**	**Forceps asymmetry index**	**Paramere length-width ratio**	**Forceps length-width ratio**
Genus	1 (NA)	-1.568	-3.061	-4.107	-0.572
Anisolabis	1 (NA)	-0.593	0.216	2.267	3.865
Gonolabis	1 (0.966)	1.427	-1.653	-8.550	1.534
	2 (0.024)	-3.802	-0.224	-1.078	2.272
	3 (0.009)	0.910	14.878	-1.398	0.568
	4 (0.001)	1.012	-2.706	5.547	2.375
Gonolabis (*G.* sp. 3 removed)	1 (0.975)	1.245	-1.720	-8.600	1.588
	2 (0.018)	-3.639	4.728	-2.290	2.055
	3 (0.007)	-2.266	-13.008	-0.513	-0.477

"Classification" refers to the grouping variable used (i.e., "Genus" refers to the discrimination of the two genera, "*Anisolabis*" to that between *Anisolabis* spp., etc.). Function refers to the individual discriminant functions generated, of which there can be a maximum of k – 1, where k is the number of levels of the grouping variable (morphospecies or genera in this case). Functions are numbered in order of the proportion of variance explained by each, which is shown in brackets. "Forceps asymmetry" is an index calculated as the sum of the absolute differences between the heights and widths of a specimen's two forceps, "Paramere ratio" is the ratio of a specimen's right paramere's length to its width, and "Forceps ratio" is the ratio of the maximum forceps length to the maximum forceps width. These three measurements were dimensionless; the basal width of the forceps was in mm.

Table S4. Uncorrected p-distances within and between earwig species on the Barcode of Life Data Systems public repository's cytochrome oxidase c subunit 1 barcodes (507–680 bp).

	N	***Chelidura guentheri***	***Forficula auricularia* A**	***Forficula auricularia* B**	***Labidura riparia***	***Nala lividipes***
Chelidura guentheri	6	0.001 (0.001)	0.020	0.020	0.019	0.020
Forficula auricularia A	558	0.231	< 0.001 (< 0.001)	0.015	0.020	0.020
Forficula auricularia B	130	0.220	0.102	0.003 (0.001)	0.019	0.020
Labidura riparia	6	0.189	0.207	0.205	0.006 (0.002)	0.017
Nala lividipes	5	0.199	0.197	0.196	0.147	0.007 (0.002)

Only records with species names attached and with sequences > 500 bp were retrieved. Interspecific means and variances are shown on the left and right of the diagonal. Intraspecific mean shown on diagonal with variance in brackets. Variances were calculated using 1000 bootstraps. N refers to total individuals retrieved. Forficula auricularia A and B refers to cryptic sibling species [1].

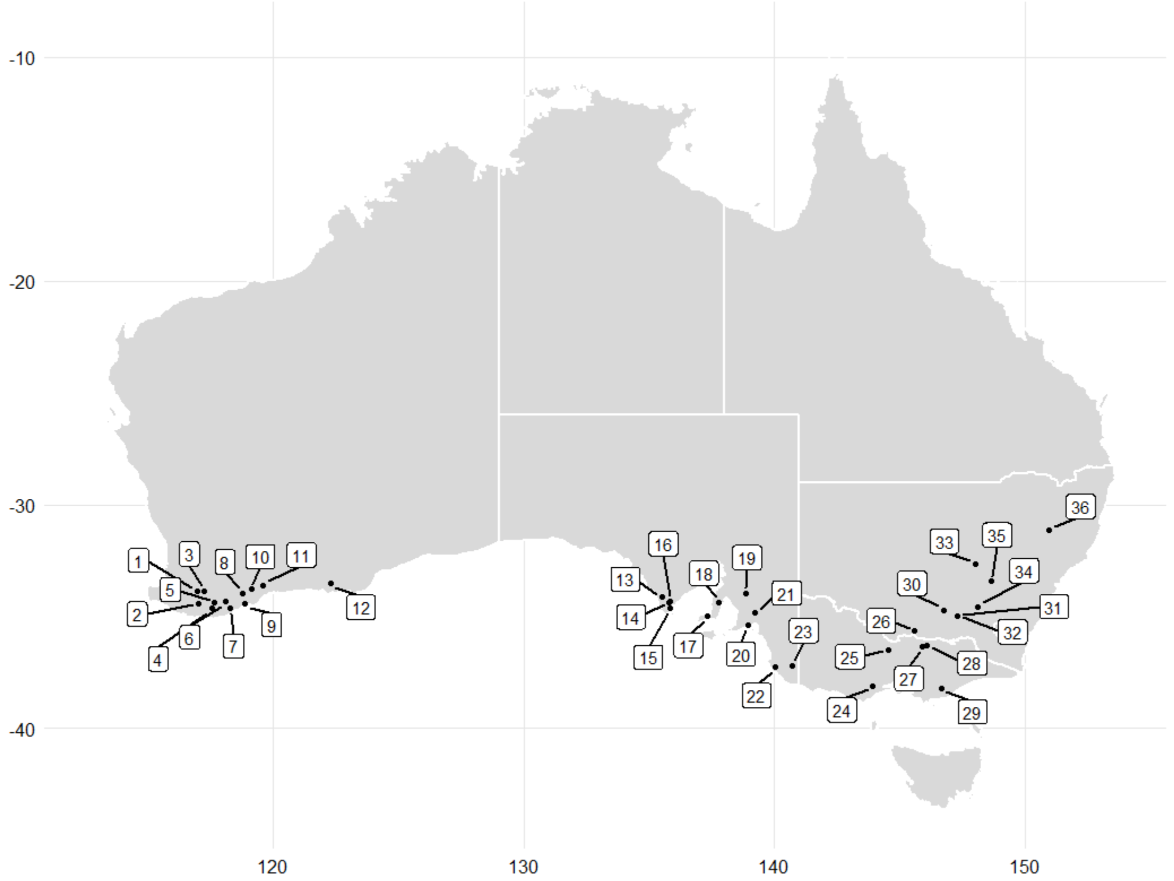

Figure S1. Map of field sites from which adult Dermaptera were collected. Numbers refer to sites listed in Table S1. Axes represent longitude and latitude.

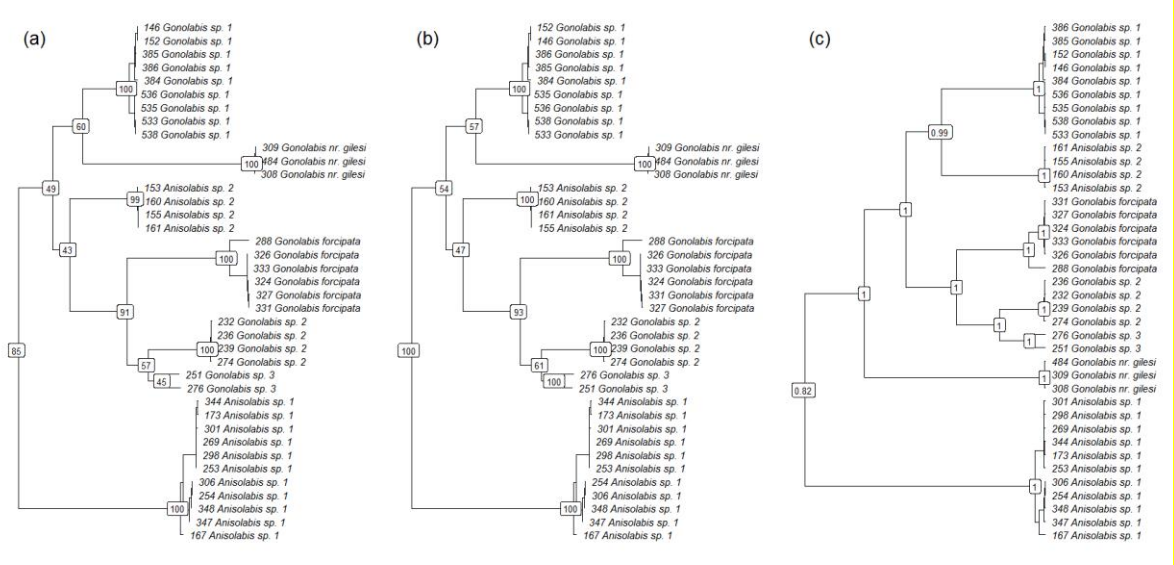

Figure S2. Gene trees inferred from alignment of mitochondrial sequence data (1016 bp) for Anisolabididae morphospecies (658 bp from cytochrome *c* oxidase subunit 1 and 358 bp from cytochrome *b*). Node labels represent bootstrap support from 1000 bootstraps (a and b) and posterior probability (c). (a) Best tree inferred under a maximum likelihood framework in RAxML using a general time reversible model of sequence evolution with a gamma-distributed rate parameter for site evolution estimated empirically; log-likelihood = −5689.987. (b) Tree inferred exactly as for (a) with a constraint tree representing the known morphospecies imposed on the search space; log-likelihood =

−5689.989. (c) Maximum clade credibility tree inferred using *BEAST, with models of site evolution estimated simultaneously to tree estimation using bModelTest and a birth-death process for the tree prior; 95% HPDI of tree log-likelihood = [−5276.756, −5254.856], mean log-likelihood = −5265.388.

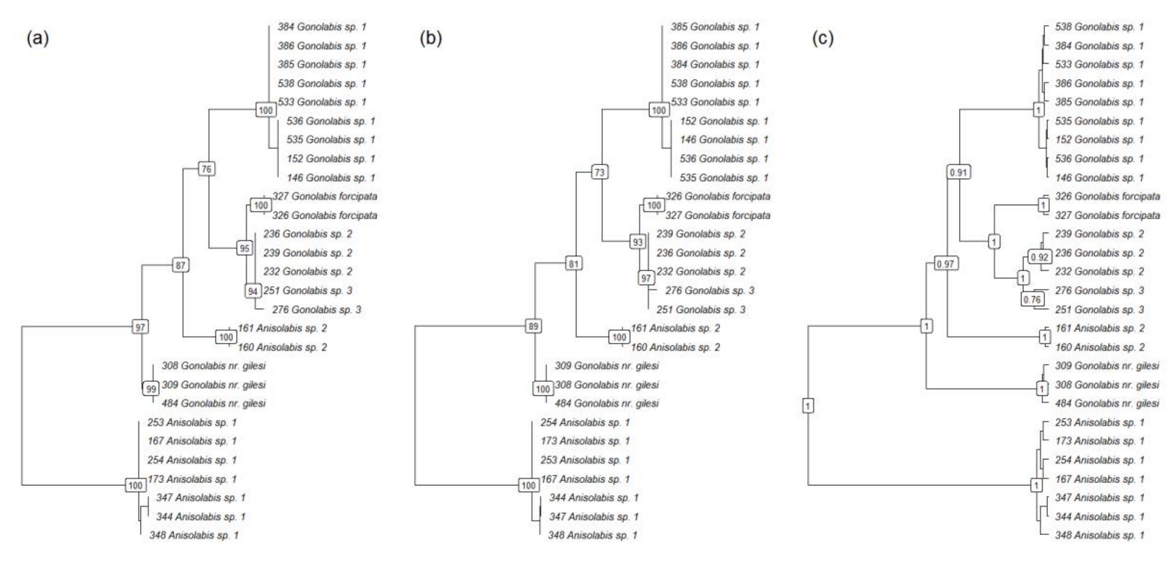

Figure S3. Gene trees inferred from alignment of a fragment of *28S* ribosomal DNA (442 bp) from Anisolabididae morphospecies. Node labels represent bootstrap support from 1000 bootstraps (a and b) and posterior probability (c). (a) Best tree inferred under a maximum likelihood framework in RAxML using a general time reversible model of sequence evolution with a gamma-distributed rate parameter for site evolution estimated empirically; log-likelihood = −1087.959. (b) Tree inferred exactly as for (a) with a constraint tree representing the known morphospecies imposed on the search space; log-likelihood = −1087.959. (c) Maximum clade credibility tree inferred using *BEAST, with models of site evolution estimated simultaneously to tree estimation using bModelTest and a birth-death process for the tree prior; 95% HPDI of tree log-likelihood = [−1123.086, −1102.800], mean log-likelihood = −1114.919.

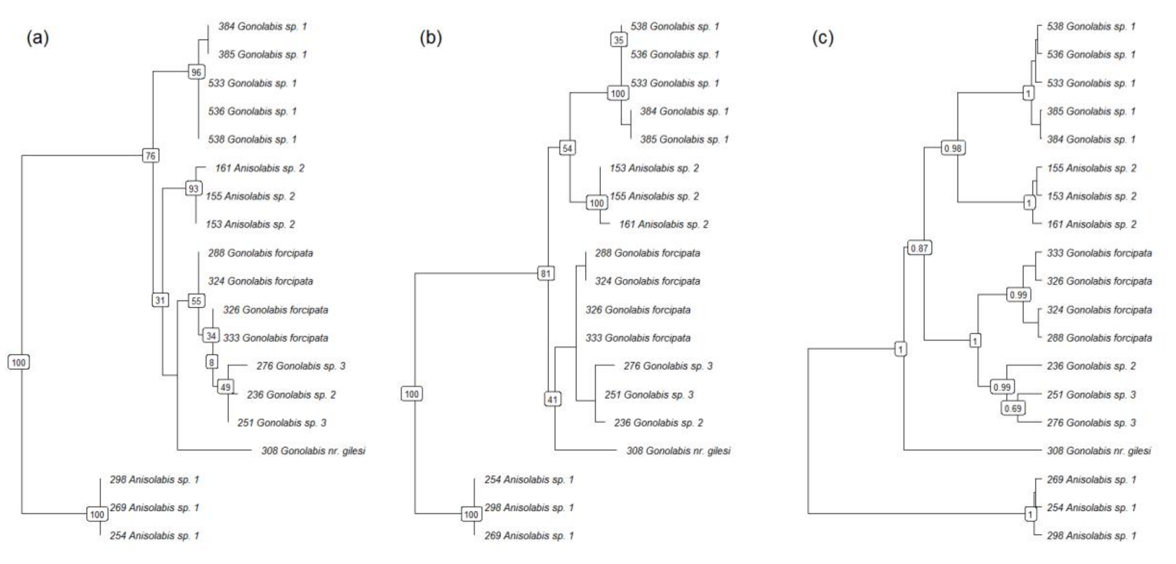

Figure S4. Gene trees inferred from alignment of a fragment of tubulin-alpha 1 (466 bp) from Anisolabididae morphospecies. Node labels represent bootstrap support from 1000 bootstraps (a and b) and posterior probability (c). (a) Best tree inferred under a maximum likelihood framework in RAxML using a general time reversible model of sequence evolution with a gamma-distributed rate parameter for site evolution estimated empirically; log-likelihood = −1183.262. (b) Tree inferred

exactly as for (a) with a constraint tree representing the known morphospecies imposed on the search space; log-likelihood = −1184.068. (c) Maximum clade credibility tree inferred using *BEAST, with models of site evolution estimated simultaneously to tree estimation using bModelTest and a birth-death process for the tree prior; 95% HPDI of tree log-likelihood = [−1086.380, −1054.702], mean log-likelihood = −1066.792.

Reference

1. Wirth, T.; Le Guellec, R.; Vancassel, M.; Veuille, M. Molecular and reproductive characterisation of sibling species in the European earwig (*Forficula auricularia*). *Evolution* **1998**, *52*, 260–265.

© 2019 by the authors. Submitted for possible open access publication under the terms and conditions of the Creative Commons Attribution (CC BY) license (http://creativecommons.org/licenses/by/4.0/).
